# Cross-Sectional Survey of Obstetrical Providers’ Knowledge, Attitudes, and Use of Noninvasive Prenatal Testing for 22q11.2 Deletion

**DOI:** 10.1055/a-2884-8604

**Published:** 2026-06-26

**Authors:** Juliana Simon, James W. Van Hook, Nabila Azeem, Madeline Allen, Hind N. Moussa

**Affiliations:** 1 Department of Obstetrics and Gynecology 89021 The University of Toledo College of Medicine and Life SciencesToledoOhioUnited States; 2 Department of Obstetrics and Gynecology 7831 University of South FloridaTampaFloridaUnited States; 3 Department of Maternal-Fetal Medicine 7924 ProMedica Health System IncToledoOhioUnited States; 4 Division of Maternal Fetal Medicine, Department of Obstetrics and Gynecology 12303 University of CincinnatiCincinnatiOhioUnited States; 5 12303 Kettering Health Maternal Fetal MedicineKetteringOhioUnited States

**Keywords:** 22q11.2 deletion syndrome, NIPT, prenatal screening, prenatal genetics, genetic counseling

## Abstract

**Objective** This study’s primary objective was to characterize obstetrical providers’ knowledge and utilization of noninvasive prenatal testing (NIPT) for 22q11.2 deletion screening.

**Study Design** A one-time survey was distributed to physicians, nurse practitioners, and midwives. Participants answered 18 multiple-choice questions pertaining to demographic information, clinical use of carrier and NIPT, and knowledge regarding use of NIPT for 22q11.2 deletion syndrome. Responses were descriptively analyzed based on years of clinical experience and provider subspecialty.

**Results** Twenty-two providers responded to the survey: six Obstetrics and Gynecology (OB/GYN) resident physicians, 10 OB/GYN generalist physicians, three Maternal–Fetal Medicine physicians, two nurse practitioners, and one midwife. All resident physicians reported never ordering 22q11.2 deletion NIPT, despite using a Trisomy screening panel, which includes 22q11.2 deletion as an opt out. Conversely, all experienced physicians ( *n* = 13) reported using expanded panels and screening for 22q11.2 deletion. Only half of our sample who completed the survey ( *n* = 10) felt adequately informed to counsel patients on screening results and procedures.

**Conclusion** Our findings demonstrate an association between years in practice and the utilization of 22q11.2 deletion NIPT screening. Enhanced educational initiatives beginning during residency are necessary to improve provider knowledge on 22q11.2 deletion screening, information regarding test ordering, and counseling procedures.

## Introduction

22q11.2 deletion syndrome is a clinically significant microdeletion syndrome associated with substantial morbidity and mortality, and which affects an estimated 1 in 2148 live births and 1 in 992 fetuses. 1 2 22q11.2 deletion syndrome encompasses conditions previously diagnosed as DiGeorge and velocardiofacial syndromes that share a common genetic etiology within the 22q11.2 locus. It represents a heterogeneous collection of multisystem symptoms, which include congenital heart defects, immunodeficiencies, cleft palate abnormalities, hypocalcemia, and neurodevelopmental delays. As such, early diagnosis is critical in establishing early intervention opportunities; management warrants a multidisciplinary approach that spans a person’s lifetime. Approximately 85% of people diagnosed with 22q11.2 deletion pathogenic copy sequence findings demonstrate LCR22A–LCR22D deletion variants that encompass the entire 3 Mb gene region. 3 Smaller, nested deletions, especially those found distally, may demonstrate overlapping symptoms with the typical deletion, but often show decreased penetrance, result in fewer congenital heart defects, and correspond to milder phenotypic features. 4 5

The advancement of noninvasive prenatal testing (NIPT) has altered how patients choose to assess fetal aneuploidy risk and has led to a decrease in invasive procedures, such as amniocentesis and chorionic villus sampling. 6 7 The introduction of NIPT for common Trisomies in national screening protocols has even presented an opportunity for early detection of other genetic conditions, such as 22q11.2 deletion syndrome. 8 Recent advances in high-throughput sequencing have led to the development of single-nucleotide polymorphism and whole-genome sequencing-based NIPT to detect sex chromosome aneuploidies and copy number variations, including microdeletion and microduplication syndromes. Continued developments have established significant improvement of NIPT performance to screen for 22q11.2 deletion syndrome with enhanced testing sensitivity and the ability to identify nested deletions typically undetectable by standard fluorescence in situ hybridization assays. However, the performance of NIPT for 22q11.2 deletion syndrome still lags behind that of the common Trisomies, with a range of 70–83% sensitivity reported, depending on the type of testing used. 9 10

The American College of Obstetricians and Gynecologists’ recently published practice advisory recommends against microdeletion screening in the general population, and specifically, states that patients who elect for 22q11.2 deletion syndrome screening should only do so after receiving comprehensive pretest counseling. 11 The American College of Medical Genetics and Genomics currently recommends offering all pregnant patients 22q11.2 screening, conditional upon shared decision-making. 12 However, there are no standardized guidelines that providers can follow regarding how to best utilize these measures. Multiple laboratories offer 22q11.2 deletion screening either as add-ons to or included with the standard Trisomy panel. For the latter, clinicians must opt out of testing if they or their patients choose. Consequently, prenatal care providers must provide education and guidance to patients regarding the complex decisions pertaining to NIPT and the results of such testing.

22q11.2 deletion NIPT demonstrates potential for universal screening benefits. Yet, the integration of 22q11.2 deletion NIPT into clinical practice remains inconsistent and largely undefined. It is important to understand what clinicians and medical trainees understand regarding 22q11.2 testing, how providers discuss the benefits and limitations of testing to patients, and how they mobilize care management. Therefore, our primary objective was to qualitatively assess the knowledge and attitudes of prenatal care providers regarding the use of NIPT for 22q11.2 deletion screening. We sought to collect hypothesis-generating data that could help identify patterns in current practices, as well as gaps in provider education and patient counseling.

## Materials and Methods

We conducted a cross-sectional survey study among health care providers from the Obstetrics and Gynecology (OB/GYN) and Maternal-Fetal Medicine (MFM) departments at a community based academic medical center and a large referral center-community hospital system in Northwest Ohio. Eligible participants included trainees, staff, and faculty members. Eligible participants were contacted via email. Initial data collection ran from April 23, 2024 through June 5, 2024; the original survey was sent again to collect more responses from September 3, 2025 through September 30, 2025.

The survey was administered using the QuestionPro software. All participants were able to access the survey via a link or QR code. Responses were anonymous, and no personal information was collected or stored. The University of Toledo and ProMedica Toledo Hospital’s institutional review boards approved this study with a partial waiver of informed consent (IRB #301809; IRB #23-112). All respondents were provided access to a downloadable copy of the consent form upon completion of the survey.

The survey consisted of 18 multiple-choice questions selected via the Delphi method. We collected demographic information, including the respondent’s specialty, years of experience, and percentage of obstetrical patients cared for in practice. We then collected information regarding the provider’s experience and use of carrier and NIPT screening. This included how often the respondent orders carrier screening, the type of carrier screening panels offered to patients, how many types of NIPT panels they offer, as well as if they use NIPT to screen for other conditions not included in the Trisomy panel, including sex chromosome aneuploidies and microdeletion/microduplication syndromes. Finally, we gathered information about respondents’ knowledge and personal attitudes regarding the use of NIPT for 22q11.2 deletion syndrome. Participants were asked to reflect on their knowledge of 22q11.2 deletion syndrome, confidence in explaining testing procedures and results, as well as ability to refer patients to appropriate care services via selecting “yes” or “no” responses. All responses were analyzed descriptively. The outcomes of interest we considered were based on factors related to experience in genetic counseling. These included respondents’ years of clinical experience, type of provider, as well as provider subspecialty.

## Results

**Fig. 1:**
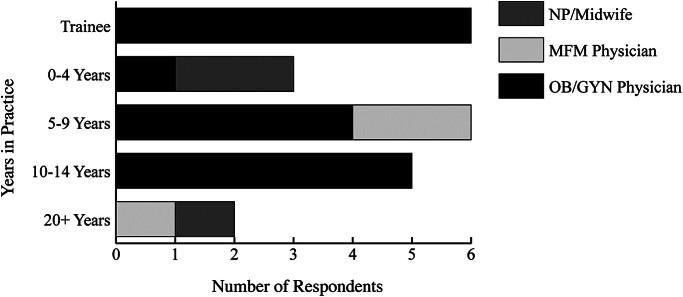
Demographic profile of survey respondents. Type of provider, specialty of practice, and years of experience among survey respondents ( *n* = 22).

Out of the 77 providers that were contacted, 22 responded to the survey, for a response rate of 29%. A total of 19 physicians completed the survey: six OB/GYN residents, 10 OB/GYN generalists, and three MFM physicians. Our sample also included two nurse practitioners and one certified nurse midwife ( Fig. 1 ). All respondents in our sample reported using NIPT for fetal aneuploidies (Trisomies 21, 18, and 13), and 95% ( *n* = 21) offered carrier screening. Our data indicated variability in the use of NIPT based on years in practice in our physician sample. As such, we analyzed result findings based on this factor. Although our sample was small, we also descriptively analyzed response differences among MFM specialists as well as nurse practitioners and midwives.

### Trainees’ Use of 22q11.2 Deletion Screening

Resident physicians reported using one type of NIPT panel for obstetric patients seen in the resident clinic. All respondents ( *n* = 6) reported never using NIPT to screen for 22q11.2 deletion syndrome; however, one trainee respondent indicated using NIPT to screen for other conditions not included in the Trisomy panel, including sex chromosome aneuploidies. Despite survey responses, the one panel offered in the resident clinic does, in fact, include 22q11.2 deletion screening with a choice to opt out of screening. Respondents were therefore unaware of ordering screening when selecting for the standard Trisomy panel, signifying a gap in knowledge. One trainee in our sample provided discordant answers, although this respondent was able to identify that the screening panel used included 22q11.2 deletion syndrome, they reported never ordering screening for their patients, despite their experience ordering the Trisomy panel.

### Experienced Physicians’ Use of 22q11.2 Deletion Screening

Conversely, all other physicians ( *n* = 13) in our sample reported using expanded NIPT panels, with 46% ( *n* = 6) selecting 22q11.2 screening as an add-on. Sixty-two percent ( *n* = 8) of experienced physicians screened all obstetric patients for 22q11.2 deletion, whereas the other respondents in our sample, including two of the three MFM physicians, selected screening only for patients who presented with a detected fetal congenital anomaly or family history positive for aneuploidy or other genetic disorder. Twenty-three percent ( *n* = 3) indicated always ordering 22q11.2 screening, whereas all MFM specialists indicated ordering screening either sometimes (50%) or often (75%). Additionally, 92% ( *n* = 12) of physicians excluding trainees offered multiple NIPT options to their patients.

**Fig. 2:**
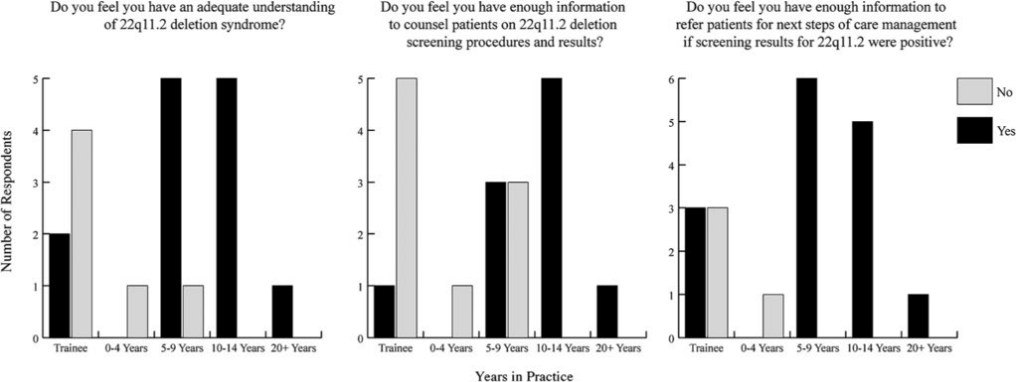
Physicians’ knowledge of screening, confidence in counseling, and next steps to care. This figure demonstrates the correlation between years of clinical experience and confidence in patient counseling among our physician sample ( *n* = 19).

Among our physician sample ( *n* = 19), physicians with 5 or more years of clinical experience endorsed greater confidence in knowledge of 22q11.2 deletion syndrome and patient referral for next steps to care compared with trainee respondents ( Fig. 2 ). Regarding provider knowledge, two OB/GYN physicians with greater than 5 years of experience incorrectly equated screening sensitivities for 22q11.2 deletion with the common Trisomies. Only half of our sample who completed the survey ( *n* = 10), which included the three MFM specialists as well as the midwife respondent, felt adequately informed to counsel patients on 22q11.2 deletion screening. Additionally, 79% ( *n* = 15) of all physician respondents indicated confidence in managing positive screening results.

### Nurse Practitioners’ and Midwifes’ Use of 22q11.2 Deletion Screening

We gathered data from two nurse practitioners and one midwife. In this sample, one nurse practitioner and the one midwife reported always screening patients for 22q11.2 deletion syndrome, despite using a testing panel that does not already include screening. The other nurse practitioner reported never screening patients. Both nurse practitioners, who indicated less than 4 years of clinical experience, did not complete the final section of the survey, and therefore did not indicate their opinions regarding their understanding of 22q11.2 deletion syndrome and confidence in counseling patients. The midwife respondent, who has practiced for more than 20 years, did not report confidence in their understanding of the syndrome, or being able to counsel patients, however, did endorse confidence in their ability to refer a patient to next steps of care, if screening resulted positive.

## Discussion

Early intervention via expanded NIPT is clinically beneficial. However, there is a lack of data regarding how perinatal providers use NIPT for 22q11.2 deletion screening in practice. Considering the increasing number of NIPT panels commercially available, our study helps to characterize patterns in screening utilization and provider knowledge. Understanding these factors can help inform future clinical guidelines and educational initiatives to enhance the utilization of NIPT in prenatal care, with the goal of improving time to diagnosis and opportunities for early intervention.

Provider knowledge of NIPT is an important factor in improving the decision-making process; however, knowledge varies significantly among specialties (e.g., OB/GYN generalist, MFM specialist, Primary Care) and type of provider counseling (e.g., physician, physician’s assistant, midwife, genetic counselor). 13 14 The three MFM specialists in our sample reported confidence in all aspects of patient counseling and management of results. Further data are warranted to characterize the extent to which specialty and type of provider ordering testing influences use of screening and counseling experiences for 22q11.2 deletion syndrome specifically.

Our findings underscore the need for enhanced education on screening procedures and results to ensure comprehensive patient counseling. Only half of our sample indicated confidence in counseling; this is significant considering all physicians’ experience with ordering 22q11.2 deletion screening. Most notably, our results indicate the importance of initiating educational opportunities during residency training. Although OB/GYN residents believe genetics to be important to the specialty, approximately half of residents surveyed in one study reported that their education in residency was not adequate to prepare them for the responsibilities of ordering and interpreting testing. 15 Educational initiatives, both experiential and didactic based, can help inform trainees of important updates in clinical genetics.

Broadly, it is important to reflect on the utilization and clinical benefits of 22q11.2 deletion NIPT when considering the prospect of universal prenatal screening. Ultrasonographic findings can prompt concern for 22q11.2 deletion syndrome in some fetuses during the prenatal period. Typical features of concern include conotruncal abnormalities, increased nuchal translucency, intrauterine growth restriction, polyhydramnios, and clubfeet. These findings can help obstetricians stratify fetuses at high risk for 22q11.2 deletion syndrome who warrant diagnostic testing, important for improving postnatal morbidity and mortality, reported as high as 14%. 16 17 18 Prenatal diagnosis can allow for delivery at a tertiary care center and results in less complications, such as failure to thrive. 19

However, not all individuals with a 22q11.2 deletion present with ultrasonographic features that will prompt expeditious diagnostic workup. Universal prenatal screening can allow for early detection and clinical management beginning in the perinatal period, even for those who would have presented with symptoms later in life. For these individuals, diagnosis relies on physicians to recognize key syndromic features on physical exam. Symptoms such as cardiac abnormalities, hypocalcemia, and palatal deformities often prompt diagnosis sooner than symptoms such as neurodevelopmental delays. 19 Additionally, non-White individuals may experience greater diagnostic delays, due in part to clinical reliance on facial dysmorphisms specifically described in white patients. 20 21 22

Considering the phenotypic heterogeneity and variable penetrance of 22q11.2 deletion syndrome, relying on physician awareness for genetic referral poses undue challenges that lead to diagnostic delays, and which prenatal screening can help offset. In a study of 202 adults with 22q11.2 deletion syndrome, the average time to diagnosis was almost 5 years, requiring families to consult an average of seven different specialists before receiving a genetic diagnosis. 20 Surprisingly, there was no significant difference when comparing the time to diagnosis in the older versus younger populations, despite the increasing clinical availability of genetic testing assays. Thus, there persists a gap in the clinical identification of presenting symptoms and referral for genetic testing that impacts time to care and which presents an opportunity for universal prenatal screening.

To optimize the clinical benefits of prenatal screening, a thought-out process regarding counseling and test choice becomes crucial for providing the best information to individual patients faced with prenatal screening decisions. Additionally, guidelines regarding management of positive and negative prenatal results become important, especially in cases of fetuses with ultrasound-detected abnormalities. A recent analysis showed that more than half of newborns who screened positive for 22q11.2 deletion syndrome did not undergo diagnostic testing at time of birth, signifying an important gap in posttest counseling and follow-up. 23 Providers must educate patients about screening test limitations, as well as prompt follow-up for prenatal ultrasound findings and postnatal diagnostic testing when prenatal diagnostic testing is deferred.

While our study focused on provider knowledge and opinions, it is equally necessary to gauge patient attitudes and understanding of 22q11.2 deletion screening as implementation of screening measures increases. While patients are aware of more common aneuploidies such as trisomy 21, they often lack knowledge of microdeletion and microduplication testing. Patients therefore undergo testing that they often do not completely understand. 24 From a fundamental standpoint, patients often desire testing. A survey study of 200 pregnant patients showed that 82% would choose to screen for conditions that would have an immediate impact on their child’s health. 25 Patients appreciate that NIPT allows for the opportunity for early intervention and to emotionally prepare for a child with a genetic syndrome, yet there is also concern for the limitations of screening and the effects that inaccurate results may have on the pregnancy experience. 26 Future studies should assess patient attitudes about 22q11.2 deletion screening specifically and the extent to which all patient populations undergo counseling about screening tests and interpretation of results.

Our initial findings help characterize how providers practically use 22q11.2 deletion NIPT; however, our results are limited in scope due to the small sample size of our study. Future studies would aim to expand upon these initial findings with a larger and more diverse cohort of providers who order genetic testing, including more nurse practitioners, physician assistants, and genetic counselors. Comparing patterns in counseling and the use of 22q11.2 deletion NIPT among various geographic locations, urban versus rural communities, and as influenced by patient sociodemographic factors would importantly build upon previous research efforts characterizing disparities in access to prenatal genetics resources. 27

## Conclusion

In conclusion, our study demonstrates that experienced providers routinely use NIPT to screen for 22q11.2 deletion syndrome. However, there is a need for enhanced guidelines on 22q11.2 deletion screening selection as well as pretest and post-test counseling offered to patients. Our findings underscore the importance of integrating enhanced education on the use of NIPT for 22q11.2 deletion syndrome screening during residency training. Continued understanding of the use and knowledge of NIPT screening for 22q11.2 deletion syndrome among all ordering providers is warranted to further advance screening protocols and standardize counseling guidelines.

## Data Availability

The datasets used and analyzed during the current study are available from the corresponding author upon reasonable request.
